# Effects of human Umbilical Cord Stem Cells and Granulocyte Colony- Stimulating Factor (G-CSF) on Carbon Tetrachloride-Induced Nephrotoxicity

**DOI:** 10.5812/numonthly.2979

**Published:** 2012-06-20

**Authors:** Yener Koc, Mehmet Sokmen, Abdulkadir Unsal, Sebnem Cigerli, Aysim Ozagari, Taner Basturk, Elbis Ahbap, Tamer Sakaci, Ayhan Dalkilic, Nezaket Eren

**Affiliations:** 1 Department of Nephrology, Sisli Etfal Research and Educational Hospital, Istanbul, Turkey; 2 Department of Gastroenterology, Sisli Etfal Research and Educational Hospital, Istanbul, Turkey; 3 Department of Biochemistry, Sisli Etfal Research and Educational Hospital, Istanbul, Turkey; 4 Department of Pathology, Sisli Etfal Research and Educational Hospital, Istanbul, Turkey; 5 Department of Urology, Sisli Etfal Research and Educational Hospital, Istanbul, Turkey

**Keywords:** Stem Cells, Granulocyte Colony-Stimulating Factor, Carbon Tetrachloride, Acute Kidney Injury

## Abstract

**Abstract:**

Background: Recently, stem cells have been used to facilitate healing in animal models of renal failure induced by acute ischemic and nephrotoxic damage. Granulocyte colony-stimulating factor (G-CSF) has been reported to stimulate stem cell mobilization from bone marrow and these cells may contribute to renal repair.

Objectives: In the present study, the effects of G-CSF and stem cell administration as monotherapy or in combination, and the relation of these effects with the duration of therapy, have been investigated in an experimental rat model of carbon tetrachloride (CCl_4_)-induced nephrotoxicity.

Materials and Methods: The fifty rats included in the study were distributed into 4 main groups, Group 1, 2, 3, and 4, and two subgroups for each group, except for Group 1. All rats received an intraperitoneal injection of CCl_4_. Then at 6 h, Groups 1, 2a, 3a, and 4a were administered saline, stem cells, G-CSF, and stem cell plus G-CSF, respectively. At 24 h, Groups 2b, 3b, and 4b were administered stem cells, G-CSF, and stem cell plus G-CSF, respectively. All animals were sacrificed 48 h after the CCl_4_ injections. Serum urea, creatinine, sodium, and potassium levels were measured from blood samples. Tissue α-glutathione S-transferase (GST) levels were also measured from renal tissues.

Results: Serum urea was reduced in all groups when compared to Group 1, but the decrease was statistically significant only in Group 3b (P = 0.04). Serum creatinine and sodium levels were similar in all groups (P > 0.05). Tissue GST levels were lower in all groups, but the reduction was significant only in Group 4a, which was administered stem cells + G-CSF at 6 h (P = 0.01). Tubular degeneration and/or tubular dilatation were the most common pathologic changes, and their incidence was similar in all groups (P > 0.05).

Conclusions: Although both stem cell and G-CSF monotherapy led to damage reduction, the effect was not significant. However, the reduced damage by the combined use of stem cells and G-CSF, particularly during the early period, was statistically significant.

## 1. Background

Despite recent developments and expansions in therapeutic choices, acute renal failure (ARF), which presents clinically with a rapid reduction in the glomerular filtration rate (GFR), still causes high mortality and morbidity (1, 2). The most common cause of the intrinsic ARF, acute tubular necrosis (ATN), is responsible for at least 40% of hospitalizations in patients with this diagnosis. Moreover, 76% of the patients hospitalized with ARF are in intensive care units (1).

The two most common causes of ATN are ischemia and exposure to nephrotoxic agents. Development of renal hypoperfusion due to extended prerenal conditions is a major cause of ischemic ATN. Morphologically, the most frequently observed changes in nephrotoxic ATN correspond to ischemic ATN. The term toxic nephropathy is used to refer to acute renal disease caused by various diagnostic agents, chemicals, and therapeutic compounds. Carbon tetrachloride (CCl_4_) is mainly used in industrial applications for producing chlorofluorocarbons but may also be used in small amounts as a solvent in agriculture and in the dry cleaning industry. Typical manifestations of the CCl_4_ intoxication are narcosis, acute hepatic necrosis, and ATN. Although the pathogenesis of CCl_4_-caused renal dysfunction is not understood in detail, renal damage was shown to develop independently from liver damage (3, 4). In experimental studies, renal damage was demonstrated to primarily occur in the proximal tubular epithelium (5).

Epithelial cells and stem cells recently started to be used to facilitate healing in animal models of renal failure induced by acute ischemic and nephrotoxic damage. Granulocyte colony-stimulating factor (G-CSF) was reported to stimulate stem cell mobilization from the bone marrow, and these cells may contribute to renal repair (5). However, the number of trials comparing the effects of G-CSF and stem cells on renal tissue is limited.

## 2. Objectives

The purpose of the present study was to create an experimental CCl_4_ nephrotoxicity model in rats, and to use this model to investigate the therapeutic effects of G-CSF and human umbilical cord blood stem cells, administered individually or in combination, and the relation between these effects and the treatment timeline.

## 3. Materials and Methods

The study was carried out in the experimental animal laboratory at the University of Marmara Faculty of Medicine (UMFM), after receiving the approval from the ethical board of Sisli Etfal Education and Research Hospital. The study used Norvegicus rattus sp. Wistar strain male rats (age: 3–6 months; weight: 250–350 g) obtained from the experimental animal laboratory.

All rats were placed under observation 7 days before the experiment in the experimental animal laboratory to ensure that they were oriented. The lab temperature was maintained at 22 ± 2 °C, and proper illumination was provided between 08.00 AM and 08.00 PM before and during treatment. The animals were kept in cages placed in a well-ventilated room, with maximum 3 animals per cage, and were given fresh tap water and standard food. Cages were maintained daily.

A total of 50 rats were studied. The rats were distributed into 4 main groups. Group 1 consisted of eight rats, and each of the remaining groups included 14 rats. All groups, excluding Group 1, were further divided into two subgroups including 7 rats each. All rats received an intraperitoneal (i.p.) injection of 0.5 ml/kg CCl_4_ dissolved in olive oil in equal proportions (1:1). Eight rats in Group 1 were administered saline injection 6 h following the CCl_4_ injection, while 7 of 14 rats in Group 2 (Group 2a) and the remaining rats from the same group (Group 2b) were administered i.p. stem cell injections (2 million cells/kg) 6 h and 24 h after the initial CCl_4_ injection, respectively. G-CSF (150 µg/kg) was administered via the i.p. route at 6 h to 7 of 14 rats in Group 3 (Group 3a) and at 24 h to the remaining 7 rats from the same group (Group 3b) while stem cells (2 million cells/kg), and G-CSF injections (150 µg/kg) were administered to 7 of 14 rats in Group 4 at 6 h (Group 4a) and at 24 h to the remaining 7 rats (Group 4b).

All animals were sacrificed 48 h after the CCl_4_ injection, after anesthesia was induced with ether inhalation. Blood samples were collected from the portal vein by bilateral perilumbar vertical incision and serum urea, creatinine, sodium, and potassium levels were assessed. Part of the excised renal tissues were placed in 10% formaldehyde solution for histopathologic analysis, and the rest was immediately stored at -80°C in liquid nitrogen until tissue analysis.

### 3.1. Tissue Analyses

Kidney biopsy samples were frozen at -80 °C. The samples were thawed to - 25°C and exposed to ambient temperature on the day of the analysis. Excess blood on tissues was washed off with physiological serum. Renal tissue samples were weighed and placed in Complete solution to obtain 30 mg of tissue sample per mL. The solution was prepared by mixing one tablet of Complete protease inhibitor (Roche) in 25 mL extraction solution and was maintained refrigerated. The function of this solution is to inhibit tissue proteases such as serine, cysteine, and metalloproteases. Tissue samples transferred into the Complete solution were first homogenized with a homogenizer and then centrifuged, and materials collected from the surface were subsequently used to study both total tissue protein and GST levels.

Total tissue protein was studied by the Lowry method. As a part of this process, the proteins were initially made to react with copper ions in an alkaline medium, followed by reduction with phosphomolybdic-phosphotungstic acid. The intensity of the resulting blue color was found to be directly proportional to protein concentrations and the protein amounts were calculated. Tissue GST levels were analyzed with the Rat Urinary Alpha GST EIA Kit (Biotrin laboratory kit Code Nº BIO64RT) and the ELISA method. The results of the tissue studies were presented as µg per gram of protein.

### 3.2. Human Umbilical Cord Stem Cells and G-CSF

After normal birth, human umbilical cord blood was collected in pediatric blood bags containing CPDA (citrate phosphate dextrose adenine), with the written consent of healthy donors. Collected blood was sent with a special courier to the umbilical cord blood bank (GENKORD- Istanbul), a stem cell research and maintenance lab, for stem cell isolation.

Samples supplied from the cord blood bank were centrifuged for 20min at 400 cycles/min. Erythrocytes in suspension were digested in a NH4Cl2 solution, at pH 7.2, and mononuclear cells were separated. Cell counts were performed using the ABX MICROS 60 (France) equipment. Cells were transferred to sterile injectors after being diluted to achieve a proportion of 5600 per µL and were sent to the animal laboratory conducting the experiment. G-CSF was obtained from a preparation with the trade name Neupogen (30 million units/vial) (Roche). Both G-CSF and the isolated stem cells were maintained at +4°C for 36 h during the experiment.

### 3.3. Histopathologic Analyses

All materials were analyzed by a pathologist blinded to both the groups and the treatments. Analyses were performed using light microscopy. Renal tissues fixed in 10% formaldehyde solution were immersed in paraffin blocks after routine tissue observations and 4 µm tissue sections were stained with hematoxylin eosin. The light microscopy analysis of renal tissues was assessed according to data from Houghton et al. (6). We evaluated the presence of tubular degeneration and/or necrosis, tubular dilatation, tubular atrophy, interstitial edema, fibrosis, and inflammation, and all the parameters were scored based on the intensity of lesions as 0 (normal), 1 (mild), 2 (moderate), or 3 (severe).

All data were uploaded to the SPSS 13.0 for Windows software. All parameters were presented as mean ± SD. The one-way ANOVA test was used to analyze inter-group parametric data. Scheffe’s post-hoc test was applied to parameters that were determined to present significant differences. The Kruskal Wallis test was used for the analysis of non-parametric data in the groups. Statistical significance was set at P < 0.05 for inter-group statistical evaluations.

## 4. Results

The biochemical parameters obtained from the blood samples collected from all the groups and the GST levels obtained by renal tissue homogenization are presented in Table 1 and Table 2 as mean ± SD. Analyses of chemical parameters (serum urea, creatinine, sodium, and potassium levels) and tissue GST levels in all the study groups were performed using the one-way ANOVA test. These analyses demonstrated differences among the groups only for serum urea and potassium levels and tissue GST levels, whereas no significant differences were detected for serum creatinine and sodium levels (Table 3). The parameters presenting significant differences, i.e., serum urea and potassium levels and tissue GST levels, were further analyzed by using Scheffe’s post-hoc test.

**Table 1:** Biochemical Parameters of the Groups

**Groups**	**Urea, mg/dL**	**Creatinine, mg/dL**	**Sodium, mEq/L**	**Potassium, mEq/L**
1 (control)	52.8 ± 7.9	1.3 ± 0.5	155 ± 5.7	7.6 ± 1.6
2a (6 h stem cell)	50.4 ± 5.9	1.3 ± 0.3	154 ± 8.1	7.0 ± 0.4
2b (24 h stem cell)	38.3 ± 4.5	1.2 ± 0.4	151 ± 6.0	5.4 ± 1.3
3a (6 h G-CSF)	46.2 ± 10.3	1.1 ± 0.4	153 ± 5.4	8.6 ± 1.5
3b (24 h G-CSF)	34.6 ± 11.6	1.2 ± 0.2	141 ± 20	6.8 ± 2.8
4a (6 h stem cell + G-CSF)	36.9 ± 8.6	1.3 ± 0.4	150 ± 9.5	6.4 ± 0.6
4b (24 h stem cell + G-CSF)	43.5 ± 6.6	1.1 ± 0.3	149 ± 6.4	6.2 ± 1.2

**Table 2:** Tissue GST Levels in the Groups

**Groups**	**Tissue GST, mcg/gr- protein**
1(control)	5.62 ± 4.76
2a(6 h stem cell)	2.47 ± 0.47
2b (24 h stem cell)	2.08 ± 0.61
3a (6 h G-CSF)	2.15 ± 1.22
3b (24 h G-CSF)	2.30 ± 1.17
4a (6 h stem cell + G-CSF)	1.11 ± 0.27
4b (24 h stem cell + G-CSF)	1.75 ± 0.35

**Table 3:** The Results of One-Way ANOVA Tests for All Parameters

**Parameters (Between All Groups)**	**F**	**P**
Urea, mg/dL	4.180	0.003
Creatinine, mg/dL	0.408	0.868
Sodium, mEq/dL	1.241	0.310
Potassium, mEq/dL	2.456	0.044
Tissue GST, mcg/gr-protein	3.858	0.004

The level of serum urea in the control group (Group 1) was lower than in each treatment group. However, the inter-group analysis showed a significant decrease only for the group that received G-CSF at 24 h (Group 3b) (P = 0.04). Although significant differences in serum potassium levels were detected among groups by ANOVA, the inter-group post-hoc analysis did not reveal significant differences. Tissue GST levels were lower in each treatment group than in the control group, but the decrease was significant only for the group treated with stem cells together with G-CSF at 6 h (Group 4a) (P = 0.01).

Tubular degeneration and/or tubular dilatation with necrosis were the most common pathologic changes observed during the histopathologic examination of renal tissues. Mild tubular degeneration and/or necrosis were detected in all the rats from Groups 1, 2a, 2b, 3a, and 4b and in 86% of rats from Groups 3b and 4a (n = 6). Moderate tubular degeneration and/or necrosis were noted in only one rat from Group 4a. The findings are presented in Table 4 as percentages that reflect the severity of the tubular dilatation. No pathology indicative of glomerular pathology, interstitial edema, or interstitial fibrosis was observed in any of the preparations during the histopathologic examination of renal tissues except for a mild tubular atrophy in only one preparation from Group 1. Mild interstitial inflammation was noted in five preparations in total (10%), one in each group, excluding Groups 3a and 4b. Analysis of the data from the histopathologic examination of renal tissues revealed no significant differences among groups in terms of tubular degeneration (and/or necrosis) or tubular dilatation (P = 0.32, P = 0.20, respectively).

**Table 4:** The Frequency of Tubular Dilatation in the Groups

Groups	Tubular Dilatation, %			
	No	Mild	Moderate	Severe
1	12.5	75	12.5	-
2a	28.5	71.5	-	-
2b	57	43	-	-
3a	71.5	28.5	-	-
3b	28.5	71.5	-	-
4a	43	57	-	-
4b	57	43	-	-

In conclusion, although the improvement in histopathologic parameters noted in the treated groups (Groups 2, 3, and 4) did not reach statistical significance, reduced damage was observed based on tissue GST levels used as an early indicator of damage; this reduction was particularly significant in the group treated with the combination of stem cells and G-CSF early on, i.e., at 6 h (Group 4a).

## 5. Discussion

Except for the current adjuvant treatments, no specific therapy exists for acute renal failure. The mechanisms underlying renal damage repair are poorly understood, but the recent consensus is that stem cells or progenitor cells of renal or extra-renal origin contribute to the repair process during the recovery from ARF by proliferating within the kidney. A popular model in this regard involves the removal of damaged or dead cells and the induction of stem cell immigration into the necrotic zone, followed by their local differentiation and proliferation (7).

Recent studies demonstrated that stem cells of bone marrow origin contribute to the regeneration of non-hematopoietic tissues. Endothelial, epithelial, and mesangial stem cells of bone marrow origin have been detected in murine kidneys following renal damage (8-10). Besides, several studies have shown that mesenchymal stem cells and stem cells of smooth muscle origin, in addition to hematopoietic stem cells, improve renal structure and function (7, 11, 12).

Alison et al. (13) first showed that stem cells of extrarenal origin (bone marrow) may differentiate into renal tubular cells. Both Poulsom et al. (14) and Gupta et al. (15) reported the presence of Y chromosome (+) tubular cells in the transplanted kidneys of males who received the organs from female donors, suggesting that extrarenal cells gradually proliferate in the tubules. The ratio of Y chromosome (+) tubular cells identified in these two studies was 0.6–6.8% and 1%, respectively. In another study on a rat model, cells of male origin were detected in female rat kidneys after ischemia (16).

A study performed by Kale. et al. also supports this moel. In this study, rats were separated into three groups (control, bone marrow ablation, and bone marrow ablation with stem cell transplantation), and stem cells were administered 2.5 h after transient ischemia. Serum BUN levels peaked on day 2 in the group treated with stem cells and returned to basal levels on day 7, and the difference was not significant compared to the control group, with the only significant reduction being noted relative to the ablation-treated group. Histopathologic examination, on the other hand, revealed that stem cells were present in renal tubular tissue 48 h after ischemia. This study showed that even though the effects of stem cells center on repair, the early course ARF in rats with no functional bone marrow was worse, confirming that stem cells of bone marrow origin also have early protective effects against damage (8).

In our study, contrary to the above studies, even though the level of serum urea in the group treated with stem cells (both at 6 h and at 24 h) was lower than in the control group, the difference did not reach statistical significance. This difference is possibly associated with the time point at which the stem cell treatment was administered.

Experimental studies have provided evidence that bone marrow cells have limited contribution in repairing damaged renal tissues, probably because of the low circulating levels of bone marrow stem cells (10). Orlic et al. showed that hematopoietic stem cells induced by cytokines before myocardial infarction significantly increased the cardiac function (17). Another study, based on the above work, demonstrated a synergistic increase in stem cell mobilization when combined with G-CSF (18). Iwasaki et al. also reported that G-CSF boosted the mobilization of bone marrow stem cells, increasing renal function recovery and reducing cisplatin-caused tubular damage (19), which was supported by similar findings by Stokman et al. in rats with ischemic renal damage (10). However, in both studies, cytokines (G-CSF, M-CSF) were administered before the induction of renal damage, and nevertheless, no significant reduction was noted in serum urea and creatinine levels measured on day 3 following the damage compared to the groups that did not receive cytokines.

Serum creatinine levels observed in the present study were similar to the findings of the above studies (non-significant reduction), even though G-CSF was administered following damage in this study. On the contrary, serum urea levels were significantly decreased in the group administered G-CSF at 24 h as compared to the control group. However, we do not believe that this difference is directly associated with the effects of G-CSF. When serum urea levels are assessed in this group together with creatinine and sodium levels and given fact that only spontaneous water requirements were met at the beginning and during the course of the present study, it is possible that the volume status of these rats may have differed from that in other groups, and that the decreased serum urea and sodium levels were due to the above-mentioned facts.

Experimental studies have demonstrated that the renal damage due to CCl_4_ occurs primarily in the proximal tubular epithelium. Degeneration, vacuolization, and necrosis develop to various extents in tubular cells, and these effects are observed within the first 2 days, particularly in studies on rats (5). Regeneration of tubular damage following ischemia or reperfusion has been shown to start in 3 days, with 50% of the tubules regenerating after 10 days (16). The complete recovery of tubular morphology takes 4 weeks (16, 20). Another study reported that tubular epithelial cell loss started 2 days following the onset of ischemia, and that improvement occurred by day 7, with complete recovery of the tubular structure taking 3 weeks (21). In the present study, plasma creatinine levels peaked at day 2 and the levels returned to baseline at day 7. Tubular damage in the present study developed as intended, but histopathologic examination did not reveal any significant differences between the treatment groups and the controls. The lack of significant differences may be associated with the early termination of the study (48 h). In other words, the present study may not have been able to demonstrate the presence of regeneration because of the short duration of the analysis. Another possibility is related to the fact that stem cell and/or G-CSF treatments were generally administered at 2 h in the available studies (8, 21), as compared to their administration at 6 and 24 h in the present study, which may have resulted in the failure to demonstrate significant effects of the stem cell or G-CSF treatments alone.

High recovery of GST from urine and the renal tissue is a short-term indicator of tubular damage induced by nephrotoxic drugs including cyclosporine, aminoglycoside, and cisplatin (22-25). Accordingly, increases observed in the control group from the present study support the idea that increases in the tissue GST levels are short-term indicators of tubular damage, with reductions in the treatment groups indicating the damage-reducing effect of the treatment.

In conclusion, the present study demonstrated that the combined use of stem cells and G-CSF is more effective than either of these treatments alone in preventing damage in the early period, and that although not demonstrated histologically, their combination contributes more significantly to renal repair.
